# Energy Landscapes of Ligand Motion Inside the Tunnel-Like Cavity of Lipid Transfer Proteins: The Case of the Pru p 3 Allergen

**DOI:** 10.3390/ijms20061432

**Published:** 2019-03-21

**Authors:** Bruno Cuevas-Zuviría, María Garrido-Arandia, Araceli Díaz-Perales, Luis F. Pacios

**Affiliations:** 1 Universidad Politécnica de Madrid (UPM), Centro de Biotecnología y Genómica de Plantas (CBGP, UPM-INIA), Campus Montegancedo-UPM, 28223 Pozuelo de Alarcón, Madrid, Spain; bruno.czuviria@upm.es (B.C.-Z.); maria.garrido@upm.es (M.G.-A.); araceli.diaz@upm.es (A.D.-P.); 2 Departamento de Biotecnología-Biología Vegetal, ETSIAAB, UPM, Ciudad Universitaria, 28040 Madrid, Spain

**Keywords:** allergy, lipid transfer proteins, molecular dynamics, enhanced sampling, metadynamics

## Abstract

Allergies are a widespread problem in western countries, affecting a large part of the population, with levels of prevalence increasingly rising due to reasons still not understood. Evidence accumulated in recent years points to an essential role played by ligands of allergen proteins in the sensitization phase of allergies. In this regard, we recently identified the natural ligand of Pru p 3, a lipid transfer protein, a major allergen from peach fruit and a model of food allergy. The ligand of Pru p 3 has been shown to play a key role in the sensitization to peach and to other plant food sources that provoke cross-reactivity in a large proportion of patients allergic to peach. However, the question of which is the binding pose of this ligand in its carrier protein, and how it can be transferred to receptors of the immune system where it develops its function as a coadjuvant was not elucidated. In this work, different molecular dynamics simulations have been considered as starting points to study the properties of the ligand–protein system in solution. Besides, an energy landscape based on collective variables that describe the process of ligand motion within the cavity of Pru p 3 was obtained by using well-tempered metadynamics. The simulations revealed the differences between distinct binding modes, and also revealed important aspects of the motion of the ligand throughout its carrier protein, relevant to its binding–unbinding process. Our findings are potentially interesting for studying protein–ligand systems beyond the specific case of the allergen protein dealt with here.

## 1. Introduction

Allergies are a major concern for health systems, particularly in westernized countries. According to data from the World Health Organization, there could be around 220–250 million people suffering from allergies [1]. However, the most alarming aspect of this disease is not only its current presence, but the steady increase in its prevalence over recent years, as suggested by large-scale studies in Europe and the United States (US) [2]. An example of this trend is the evolution of children’s hospitalization in the US due to food-induced anaphylaxis, increasing from 0.6 children per thousand hospitalizations in 2000 to 1.26 in 2009 [2]. Allergies are not only a threat to affected individuals, but also to the sustainability of the public health systems, due to its elevated cost that reaches the levels of seasonal influenza [3].

An allergy is a disorder in which an innocuous substance is perceived as dangerous by the adaptive immune system, which triggers a number of defense processes that include strong inflammatory responses, which can lead, in the worst of cases, to death itself. An allergy develops in two steps: the first sensitization phase occurs when the patient is first exposed to the allergen and his/her immune system acts by developing memory cells that produce specific IgE against the allergen, and the effector phase occurs when the patient is exposed again to the allergen and the specific IgE acts by recognizing it and activating the inflammatory response by the secretion of different substances through mastocytes and monocytes. Details about the onset of allergies can be found in [4]. There are still many gaps in the knowledge related to the origins of allergies and its prevalence. The above-mentioned increase in the prevalence of allergies could be linked to many hypothetical factors, but there are no conclusive data. While many details are well-known at the molecular level for several events in the sensitization phase and a great part of processes in the effector phase, the knowledge on the very initial stage of sensitization is scarcer. In particular, although there is information about the presentation of allergens by antigen-presenting cells (APCs) to allergen-specific T-cells, essential molecular details on the recognition of allergens by the receptors of APCs are still largely unknown.

**Figure 1:**
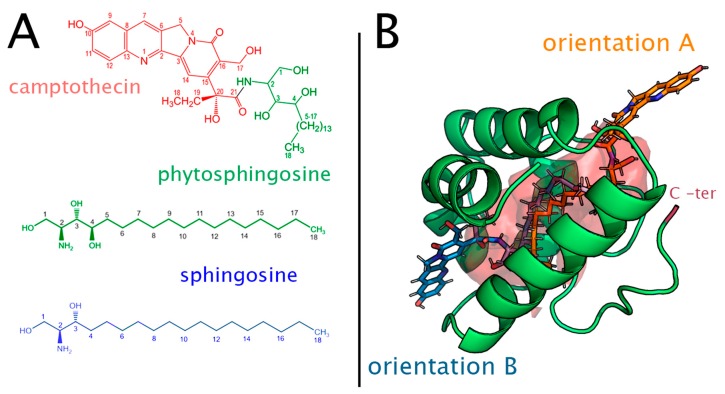
(**A**) Structural formulas of the ligands addressed in this work: the natural ligand of Pru p 3 (formed by the union of phytosphingosine and 10-hydroxy-camptothecin); phytosphingosine, and sphingosine. (**B**) Two possible orientations for the location of the ligand in the hydrophobic cavity (tunnel) of Pru p 3. The surface colored in red encloses the volume of a constant pocket inside the protein, obtained using MDPocket over molecular dynamics simulations.

In the case of food allergies, most of the research has focused on allergen proteins that elicit sensitization [5]. However, protein families have non-allergenic members without showing any special feature different from the other allergenic members, so it is yet unclear what makes a protein an allergen [5]. The observation that, within protein families characterized by the abundant presence of allergens, the transport of lipidic molecules is a relatively common feature, has led in recent years to the suggestion that those ligands could play a crucial role (probably as coadjuvants) in the allergenicity of their binding proteins [6,7]. In this regard, one example that is particularly important in food allergies is the lipid transport proteins (LTP) family, which are the proteins in charge of the transport of lipidic molecules inside their large hydrophobic cavity. In general, LTPs show a low sequence identity rate across the family and a large structural similarity [8,9,10,11]. All experimental structures of LTPs share a common compact four- α-helix fold, stabilized by four conserved disulfide bonds. The helices are aligned in parallel with the cavity of the protein, which can be also considered as a tunnel, since it is open on both sides. Experimental structures of lipidic ligands with polar headgroups inside the tunnel of several LTPs have been found in two different poses: one with the polar head located near the more hydrophilic opening of the tunnel and oriented towards the C-terminal (herein “orientation A”), and the other with the polar head oriented towards the more hydrophobic opening of the tunnel (herein “orientation B”) [12,13,14] (Figure 1).

Because LTPs are widespread in the plant kingdom and the allergenicity of some of them has long been known, they have been intensively studied [12]. Despite this extensive work, their functional roles are still obscure. LTPs have been related to many different processes, such as the deposition of waxes in the plant cell wall, the release of defense compounds [15], possible drug delivery agents [16], and biosensors of lipid molecules [17]. Traditionally, LTPs have been considered as non-specific lipid carriers, and most of the research about them has addressed different ligands to compare features with their uptake rate. Moreover, crystallographic structures of LTPs have been obtained with different lipidic ligands, leading to the generalized idea that these proteins have no specific ligands. However, we recently discovered that, at least in the case of peach (*Prunus persica)* (Pru p 3), the LTP responsible for the food allergy to this fruit is naturally bound to a specific ligand (see below, and Figure 1A). According to our research, this ligand would participate decisively in the sensitization process, acting as a crucial coadjuvant that directly interacts with receptors of the immune system [18]. Together with previously existing evidence pointing towards the importance of ligands in allergy, this finding moves the focus from the protein as the only allergenic agent to the protein–ligand complex as the key system in the sensitization phase. In other words, instead of only considering protein recognition, it seems likely that the very first stage of this phase is actually a multifactorial process, in which ligand transfer and delivery to receptors could be major events.

The natural ligand of Pru p 3 was recently identified [18] as a molecule composed of a phytosphingosine 18-C hydrophobic tail and a hydroxyl derivative of the alkaloid camptothecin, making the ligand a compound with an overall polar head-nonpolar tail structure (Figure 1A). Phytosphingosine is a major component of plant membranes, where it plays a similar role to that of sphingosine in animal and fungi cells (sphingosine is rarely found in plants) [19]. The camptothecin moiety could act as a defense compound in the plant due to its known capacity to inhibit topoisomerase I, an enzyme that removes DNA supercoils during transcription and DNA replication [18]. As for the allergy response to peach fruit, it has been shown that this natural ligand of Pru p 3 is located inside CD1d, the receptor responsible for the presentation of lipid antigens to the T-cell receptor (TCR) of invariant natural killer T (iNKT) cells, which in turn leads to the activation of the immune system against foreign lipid agents [20]. Experiments have shown that Pru p 3 without its ligand has a very low capacity to activate the immune system, and also that the ligand is necessary for the CD1d-TCR interaction that triggers the immune response. It has also been shown that camptothecin on its own does not elicit sensitization pathways but that phytosphingosine does, thus becoming an interesting target in the sensitization pathways [20].

However, essentially nothing is yet known about the process of ligand transfer and delivery to proper receptors and about the origins of the ligand specificity, a feature which, given the experimental evidence of lipid uptake in vitro, seems to be biological. In this regard, finding the right binding pose (orientation A or B, Figure 1B) is important to understand membrane-LTP interactions and LTP-CD1d ligand transfer and delivery processes, but also to understand other interesting properties, such as the protein–ligand binding free-energy, or how lipid binding could affect proteins that are generally stable. Some studies have focused on the dynamics of ligands in LTP molecules, from both theoretical and experimental points of view. Shenkarev et al. [10] used NMR to assess the binding pathways of lipid ligands to the lentil LTP (very similar in many structural details to peach LTP) and interesting results on ligand movement and interaction with LTPs were obtained [10]. Other reports have approached LTP-ligand systems from a theoretical point of view using computational tools. In Lai et al. [21], molecular dynamics (MD) simulations in the LTP of rice were performed to analyze changes in the protein mobility according to its binding to ligands. In Pacios et al. [14], the binding of different ligands found in experimental structures of plant LTPs was studied using Poisson–Boltzmann electrostatic potentials and empirical free-energy estimators in both A and B orientations. Smith et al. [22] complemented the latter work by performing MD calculations in maize and barley LTPs using different poses with the experimental ligands, suggesting that orientation A is favored. In Shi et al. [23], a computational study of a new LTP discovered in *Peganum harmala* (a medicinal grass) with possible therapeutic properties was performed using docking and MD. In Toushesh et al. [24], a docking study of different lipids was performed on a homology-based built model of a type-2 LTP of rice.

Molecular dynamics provides useful tools in studies of molecular systems. However, conventional MD suffers from two main limitations: (i) The high computational cost, and (ii) The insufficient exploration of out-of-equilibrium states. For instance, ligand unbinding in a protein–ligand complex is a process in which the ligand has to explore different non-equilibrium states to search the path from bound to unbound states (or vice-versa). For this reason, the large family of enhanced sampling methods has been developed. Metadynamics [25] is one of these techniques and is based in a history-dependent potential in the form of a sum of gaussians that is added to the potential in order to allow the exploration of new states of collective variables, which are additional degrees of freedom that can describe the interesting features and events of the molecular system [26,27,28,29,30,31]. This algorithm has proven useful to reproduce free energy landscapes according to the collective variables. However, in its initial implementation it showed convergence issues that were solved by the introduction of the well-tempered metadynamics algorithm, in which the potential of the gaussians added decreases with time to avoid oscillations between basins [32]. An interesting use of enhanced sampling tools is to elucidate the binding–unbinding paths along which ligand and proteins interact, as well as their kinetic properties as a result of the underlying energy landscape [33].

In this work, we carried out a computational study of the interaction of Pru p 3 with three related ligands with biological significance, and then we conducted a set of enhanced-sampling simulations to study the movement of the natural ligand of Pru p 3 inside the protein. In the first stage, we explored the binding between Pru p 3 and its natural ligand, phytosphingosine, and sphingosine. As mentioned above, this natural ligand seems to be a key coadjuvant in the allergic sensitization, and phytosphingosine is a component of that ligand that also acts as coadjuvant on its own. We hypothesized that cleavage of the ligand could give place to a decrease of affinity or even ligand unbinding. Sphingosine was also considered in the study to test if a very similar compound, which is absent in plant membranes but is very abundant in animal and fungi [34], could display similar dynamic features to phytosphingosine and to the natural ligand of Pru p 3. In the second stage, we used the data from previous simulations to establish collective variables (CVs) appropriate to the dynamic problem addressed, i.e., the motion of the ligand inside the tunnel-like cavity of the LTP, and then simulated this motion in terms of those CVs using the well-tempered metadynamics algorithm to obtain an energy landscape. Finally, a detailed analysis of basins and transition states was achieved to explain the features of both poses (orientations A and B) and how they could affect the ligand transfer process. While the overall methodology employed in this work is standard, the application of MD tools and analyses to the study of ligand binding at alternative orientations in the important family of LTPs has not been (to the best of our knowledge) reported before. We also believe that this study could be of interest to address other lipid–transport proteins that also feature internal tunnel-like cavities and to address the binding–unbinding dynamics of carried molecules in protein–ligand complexes. Ultimately, this work could provide a piece of information about the release of ligands, which might be useful in the complex set of molecular interactions associated with the initial stages of allergy sensitization.

## 2. Results

### 2.1. Molecular Dynamics

Molecular dynamics simulations are the starting point to understanding the dynamic properties of the system, and in this work, also the first step to setting up the process studied in terms of collective variables. Simulations were performed for the complexes of Pru p 3, with each of the three ligands presented in the introduction (the natural ligand of Pru p 3, phytosphingosine, and sphingosine, Figure 1A) in the two possible orientations A and B (Figure 1B). Although it will be further explained below, the main reason for choosing these three ligands is that they are able to characterize the dynamics of (i) a special molecule for which a computational study has not yet been done (the ligand of Pru p 3); (ii) the main hydrolysis product of the natural ligand, which could also play a role in the activation of the immune system (phytosphingosine), and (iii) a molecule similar to the hydrolysis product, whose abundance in animal cells is larger than in plants (sphingosine).

**Figure 2:**
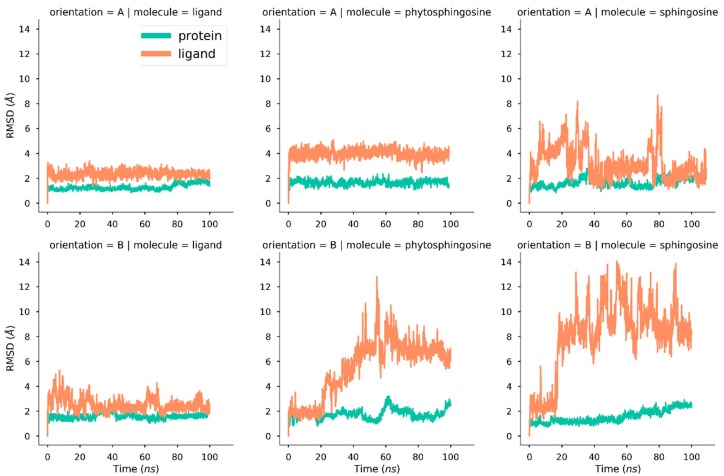
RMSD of protein (backbone atoms) and ligands along 100 ns trajectories for the orientations A and B inside the cavity of Pru p 3 of the three ligands studied: the phytosphingosine tail of the natural ligand of Pru p 3 (“ligand” label), phytosphingosine, and sphingosine.

Root mean squares deviation (RMSD) values computed along these simulations show a significantly different behavior of the molecule harbored in the protein cavity (Figure 2). The phytosphingosine tail of the natural ligand of Pru p 3 inside the cavity displays a rather stable trajectory in the two poses, although the camptothecin segment remains largely exposed to the solvent and therefore shows a larger mobility thanthe aliphatic chain. A transition found in the RMSD curve for the complete ligand in orientation B at ~40 ns corresponds to a torsion around the segment that links the camptothecin polar moiety and the phytosphingosine tail (see Figure S1). The stability of this tail inside the tunnel-like cavity, as well as that of the complete protein structure, is consistently found in three replicates of 100 ns MD trajectories (see Figure S2). The Pru p 3-phytosphingosine complex exhibits a different stability depending on whether the aliphatic chain is in orientation A or B. While in the former case, the complex is very stable with steady RMSD values of phytosphingosine about 4 Å, in the latter case RMSDs are greater than 8 Å from ~40 ns. The variations in the Pru p 3-sphingosine complex are again different in the two poses but whereas the RMSD of this chain is < 6 Å for most of simulation time in orientation A, it is > 8 Å from ~20 ns in orientation B. In any event, the three complexes in the two orientations show rather stable trajectories for the protein which, in all cases, has RMSD of backbones below 2 Å (Figure 2).

In order to obtain a picture of the most relevant residues in the protein–ligand interaction, we applied a direct procedure for clustering residues, based on specific covariance of protein–ligand contacts along the MD trajectory. In the orientation A, a relevant set of four residues was found to interact with the part of the natural ligand that joins the polar headgroup and the aliphatic chain: Asn35, Arg44, Ile77, and Pro78. For the orientation B, residues Leu10, Ile14, Val17, Leu 51, Leu54, Ser55, and Ala 66 were found to tightly interact in a similar interaction mode. Most of these residues are non-polar and are located inside the cavity, which likely indicates a dispersion-driven protein–ligand interaction. The location in the structure of Pru p 3 of both clusters of residues are shown in Figure S3A,B.

### 2.2. Protein–Ligand Binding Free Energy

Protein–ligand binding free energies were estimated by means of the MM–PBSA (Molecular Mechanics–Poisson–Boltzmann Surface Area) approach to compute ΔH values, using the Principal components analysis (PCA)-histogram method to compute ΔS values. Even though both procedures suffer from well-known limitations, they provide a feasible way to obtain reasonable values of ΔG from MD trajectories, as it is widely recognized in the MD community. To obtain an estimate of the variance of the method, these energy calculations were repeated over similar intervals of the trajectories starting at different times (Table 1). For both the natural ligand of Pru p 3 and sphingosine, orientation B is clearly more favorable than orientation A, with a difference of energy that, in the case of the natural ligand, translates into ~600-fold affinity. Phytosphingosine is different not only in that orientation A is more favorable than orientation B but also in that the affinity in the less favorable mode falls much more noticeably with respect to the favorable one.

To assess the reproducibility of MD results, three replicates of 100-ns simulations were performed for the complex of Pru p 3 and its natural ligand. Binding free energies, enthalpies, and entropies were again estimated for this complex after these three repeats. The results are gathered in Table S1 and illustrated in Figure S4 in the supplementary material.

The initial and final structures after the 100-ns MD simulations for the complexes of Pru p 3 with the three ligands in the two orientations A and B are shown in Figure S5.

### 2.3. Collective Variables of Ligand–Protein Motion

Ligand movement within a protein cavity is a process that involves all atoms, and thus all possible degrees of freedom and of movement of the system are, in principle, involved. However, most of these degrees of freedom are not relevant to analyze the motion of the ligand inside the cavity of LTPs, i.e., they are related in manifolds with lower dimensionality. Therefore, it is convenient to set an ensemble of variables dependent on the atom coordinates that can explain most of the variance of the process. These variables were defined in the conceptual framework of collective variables, where variables are defined as arbitrary functions of coordinates like the distance between groups of atoms, their angles, and others.

Pru p 3 and most of the LTPs have a tunnel with a clear entrance and an exit, regardless of the chosen orientation. Therefore, a simple assumption is that the ligands can move in an axis that is longitudinal to the tunnel, the diffusion axis. To determine that axis, we performed a pocket analysis along the trajectory of the complex of Pru p 3 and its natural ligand in orientation A, using MDPocket [35] software. A 3D tensor that specifies the frequency at which each point of space becomes a pocket was obtained from the analysis. Bins of that tensor were converted to 3D coordinates, and those coordinates were orthogonally decomposed using a treatment equivalent to a Principal Component Analysis (PCA), thus obtaining three orthogonal axes and their associated variance. The axis associated with the largest variance was selected as the diffusion axis.

**Figure 3:**
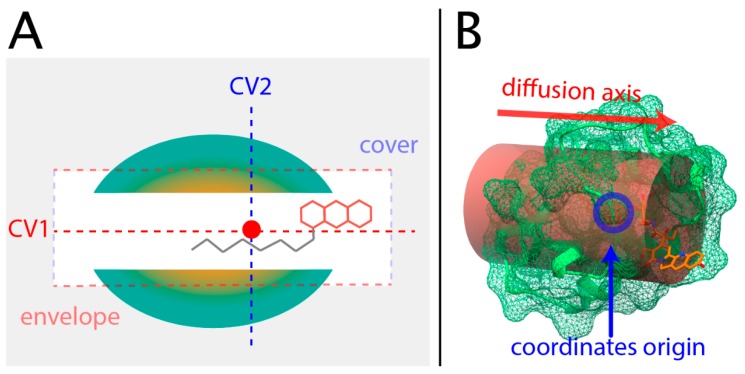
(**A**) Scheme of the collective variables (CVs) setup. (**B**) Rendering of the volume in which the ligand tail is confined using harmonic walls (see the text in Section 2.3).

Finally, two CVs were defined from the distance between atoms of the aliphatic chain and their reference starting position: the CV1 is the distance projected in the main diffusion axis, while the CV2 is the distance projected in the orthogonal plane to the CV1. Both CVs are represented in Figure 3. The “cover” and “envelope” of that figure represents the constrained region that these variables allow.

**Figure 4:**
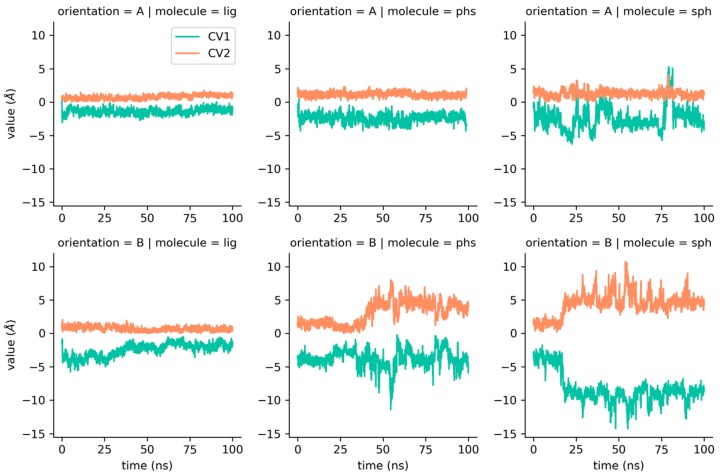
CV1 and CV2 along the unbiased molecular dynamics trajectory for the three ligands in orientations A and B calculated only for aliphatic carbons. “Lig” = natural ligand of Pru p 3, “phs” = phytosphingosine, and “sph” = sphingosine.

The values of these CVs are plotted in Figure 4. For the natural ligand of Pru p 3, the CVs indicate very stable trajectories with nearly no relevant changes. Phytosphingosine in orientation B exhibits a larger variance of CVs than in orientation A, which is consistent with the higher RMSD variation seen in Figure 2. Sphingosine in orientation A shows a steady CV2 value (lateral diffusion) but significant changes in CV1, whereas the orientation B displays large variations in both variables. In any case, CV1 values reflect that no unbinding events are suggested during the simulations.

### 2.4. Free Energy Landscape

Ligand motion inside the protein takes place in the regions and paths that are energetically possible and favored. Therefore, it is interesting to describe the energy landscape using a simplified representation of the process of interest in terms of the collective variables presented in the preceding subsection. The well-tempered metadynamics approach was used to explore the relevant regions of the collective variable CV1, which is the axis in which ligand diffusion should prevail. To avoid extending the movement of the ligand outside the protein in the solvent, a set of harmonic walls were included, imposing restraints over the values of CV1 and the CV2 to confine the ligand in the tunnel region. A rendering of this volume is depicted in Figure 3B.

**Figure 5:**
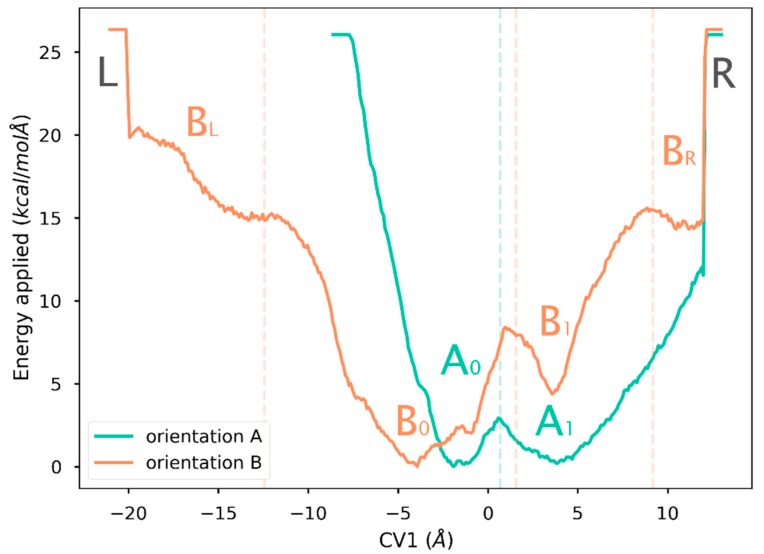
Free energy landscape obtained by well-tempered metadynamics simulations along the collective variable CV1. Dotted lines mark the different basins detected.

**Figure 6:**
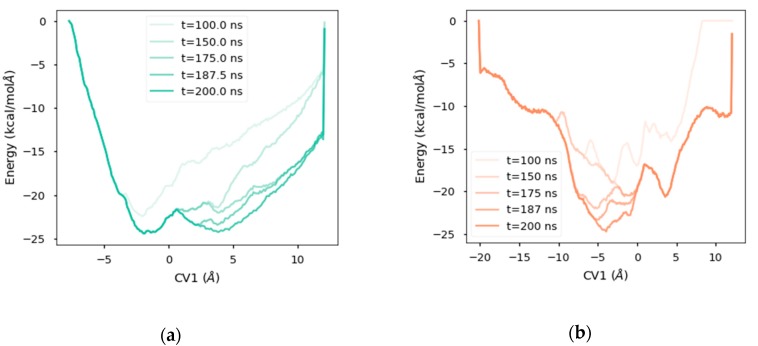
Convergence of metadynamics simulations illustrated as energy deposition along time in the diffusion axis. Latest contributions added little energy to the landscape. (**a**) Energy landscape for the orientation A. (**b**) Ibid. for the orientation B.

As was mentioned above, the metadynamics bias works by adding a potential applied over the collective variables studied (in this case, CV1) in the form of a set of gaussians. In the well-tempered metadynamics approach, the amount of energy added is gradually reduced as long as the underlying free energy landscape is filled, providing (i) more stable simulations, and (ii) a way to measure the convergence of the resulting energy landscape when energy deposition stays steadily around zero for a long period of time. This decrease is controlled by a tunable parameter, the bias temperature (ΔT). High bias temperatures allow faster explorations of the free-energy landscape, but might produce artifacts, such as protein unfolding. On the other hand, low bias temperatures may limit exploration of the free energy landscape. When this bias temperature is too low, unbiased molecular dynamics are recovered. In this work, different bias temperatures ΔT were tested, and ΔT = 4500K was found to be the best value to explore features of this system while allowing exploration of relevant regions, including possible ligand–unbinding events without disrupting the protein structure. The resulting free energy landscape is illustrated in Figure 5, and the convergence of the method as described by the decrease of the energy added over time is illustrated in Figure 6.

The plots in Figure 5 show large differences between the landscapes of orientations A and B. The landscape of orientation B was completely sampled in the allowed region (from −20 Å to +12.5 Å along the CV1), whereas the region from −20 Å to −5 Å in the landscape of orientation A was not sampled at all. This result indicates that to access that region, a much larger amount of energy than that provided in those simulations is required.

To further clarify this discussion, the next point is to examine the different regions of the free energy landscape of these simulations. The deepest basins of potential are labeled as A_0_ and B_0_ (Figure 5). Both basins are located around the reference position, which happens to correspond to the output coordinates of a docking method to prepare initial geometries of protein–ligand complexes (data not shown). The other basins, with immediately higher energy in each landscape, are labeled as A_1_ and B_1_. These two basins have different energies, although the locations of both minima are about 4 Å in the CV1 coordinate. The left and right margins of the energy landscape are labeled L and R, respectively, in Figure 5. Finally, the regions in the landscape of orientation B located between the margins and the major basins are labeled as B_L_ and B_R_ according to their closest margin. These regions are markedly differently to basins B_0_ and B_1_, but they remain partially unexplored due to the constraints along the diffusion axis. In the landscape of orientation A, there are no such basins.

### 2.5. Analysis of the Diffusion Process

Energy landscapes for ligand diffusion were characterized using a method that adds a potential which is applied to the degrees of freedom of the system. As a consequence, energy applied to allow ligand diffusion might be producing conformational changes in the protein, so that understanding these changes could be helpful to elucidate the requirements of ligand diffusion.

One of the main issues regarding ligand diffusion in a protein–ligand complex is the volume of the pocket throughout which the motion occurs. Since the natural ligand of Pru p 3, the main objective of our study, has a large headgroup with aromatic rings and polar substituents (Figure 1A), it is very possible that the movement along the diffusion axis could find steric hindrances unless conformational changes took place by increasing the protein volume. In this regard, we analyzed three relevant aspects over the trajectories: (i) the volume of the internal cavity, (ii) the volume of the protein, and (iii) the number of contacts between ligand and protein.

**Figure 7:**
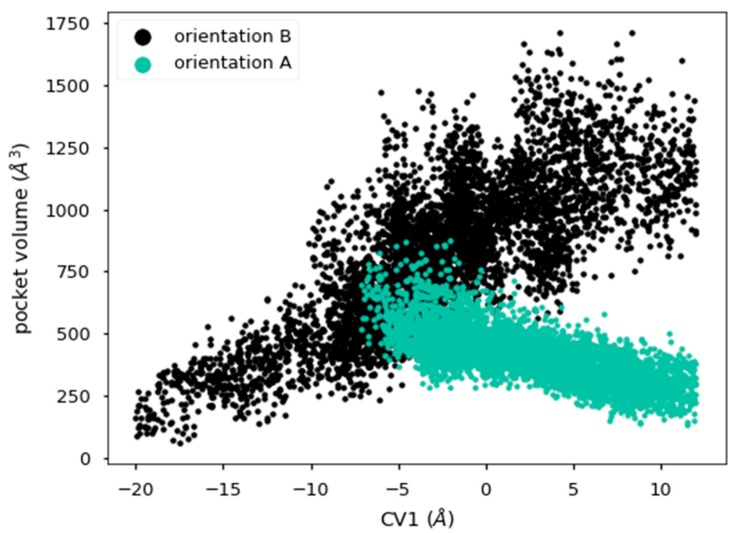
Cavity volume for each position of the ligand along the diffusion axis during metadynamics simulations. Color of orientation A was changed for visualization purposes.

The volume of the internal cavity was measured using PocketAnalyzer [36] for each frame of the molecular dynamics trajectory and is represented in terms of collective variable CV1 in Figure 7. There is a very clear correlation between the size of the cavity and the position of the ligand along the diffusion axis, as the sign of that correlation is just the opposite for each of the two orientations considered in our study. It may thus be concluded that the movement of the polar head of the ligand produces an enlargement of the internal tunnel in the protein. Moreover, when the ligand is partially unbound at both ends of the CV1 scale (Figure 7), the cavity shows a noticeable shrinking, suggesting that ligand binding is essential to keep the cavity open.

Protein size was indirectly measured using the radius of gyration computed with the alpha carbons of the protein, which is the mean distance between all these atoms and the center of mass. Our results suggest that not only the protein tunnel, but also the protein itself, enlarges when the large polar head of the ligand gets inside the tunnel, and they both reduce when the ligand partially gets out of the cavity (Figures S6 and S7). Finally, the number of contacts between the protein and the ligand was also determined in order to assess whether or not unbound ligands keep some link with the protein at the ends of the diffusion axis. Results, represented in Figure S8, show that, as expected, the number of contacts largely decrease, but they do not fall to zero, a finding that indicates that the ligands are never completely unbound.

## 3. Discussion

LTPs represent an important family of proteins which participate in many processes in plant molecular biology, as well as in human health. They have well-known common features: (i) a conserved four-helical 3D structure characterized by a hydrophobic tunnel [8,9,11,37], (ii) a lack of in vitro ligand specificity [10,16], and (iii) a ubiquitous presence in the terrestrial plants [38]. However, their role is still controversial. According to literature, LTPs would act as part of the immune system of plants by providing antimicrobial [15,39,40] and even antiviral protection [41]; they would act in the metabolic transport of lipids, especially for deposition of waxes in the cuticle [42]; and they would act in the regulation of fertilization [43], apoptosis [44], and symbiosis [45]. In principle, it should then be difficult to understand how such small proteins (~9 kDa) with a highly similar 3D structure might be able to perform such a variety of activities. The discovery of a specific lipidic ligand bound to the LTP from peach, which is besides the major allergen of this fruit (Pru p 3) [18], opens a new path to answer this question: LTPs could bind to different ligands to play each of those different roles.

Focusing on the interaction between Pru p 3 and its natural ligand recently identified in our group [18] which can be taken as an example of this new paradigm, there are two main questions about the structure of the complex: the ligand pose and the diffusion path. The first one could be solved from an energetic point of view by analyzing the differences of free energy between poses, and assuming that the one with the lowest energy will be the prevalent one. As presented above, the conformation of the natural ligand in its complex with Pru p 3 allows for two different orientations when it is harbored in the tunnel-like cavity. But there is no easy way to connect both conformations, since it would require either ligand unbinding in one pose followed by ligand binding in the other pose or a change of orientation inside the cavity, a difficult possibility in the narrow space of the LTP tunnel. Moreover, the biological evidence suggests that the ligand uptake could happen in the interface between water and cell membranes [18], so the right binding pose would in turn be dependent on the membrane-interaction mode. Therefore, a simultaneous understanding of both ligand poses and diffusion paths is needed to analyze the behavior of the complex. In this regard, a related question is the specificity of the ligand. It has customarily been assumed that LTPs were able to bind almost every kind of lipids, and a large number of studies confirm that LTPs can bind in vitro a large series of lipids, especially fatty acids of around 16C–18C [10]. It therefore seems clear that the origin of ligand specificity might be biological.

Our main objective was to understand the movement of ligands inside the protein; thus, a study of the energy landscape of motion was considered crucial. Obtaining this landscape requires working with a reduced set of coordinates (collective variables) that accurately describe the ligand diffusion inside the protein. Setting those variables demands not only a conceptual approach about the nature of the process but also information about the dynamic behavior of LTP and the binding energy of the protein in complexes with different (but related) ligands. Together with the natural ligand of Pru p 3, which has been found to be a key component in the process of allergic sensitization to peach [20], we considered phytosphingosine and sphingosine, two primary components of sphingolipids that only differ in the presence of a hydroxyl group in position 4 (Figure 1A) that are direct possible products of pH-dependent cleavage of the ligand of Pru p 3. Both are bioactive lipids which play key roles in a large variety of cellular processes (the reader is referred for example to refs. [46,47,48] and references therein) and they have been reported to bind to LTPs in vitro [16].

RMSD computed in MD simulations for the Pru p 3 protein in complex with those three ligands (Figure 2) displayed different behaviors according to the ligand type and orientation. Sphingosine exhibits higher mobility, which can be associated with the missing hydroxyl group and its missing associated capacity of putative hydrogen bonding with the protein. On the other side, the natural ligand of Pru p 3 shows a stable trajectory with shifts associated with torsions between the rigid camptothecin segment and the aliphatic chain (Figure S1), a detail not relevant in this study. Phytosphingosine dynamics are between those of the other two ligands: phytosphingosine in orientation A reaches a steady state that remains constant during the whole simulation, while in orientation B it shows an increased mobility (Figure 2 and Figure S1). Missing interactions of camptothecin with the entrance of the tunnel may explain the differences in affinity to the protein between the natural ligand and phytosphingosine in orientation B (Figure 2 and Figure S1), since the entrance of the tunnel in this orientation has a low content of polar residues. Hence, it is possible that the head of the natural ligand interacts with this region of the protein, protecting it from the solvent.

A diffusion axis, which could explain most of the ligand movement, was obtained using pocket prediction data over the MD trajectories. From that axis, two collective variables were defined using different projections of distances between ligand position and its reference state—CV1, using the projection on the diffusion axis, and CV2, using the projection orthogonal to the diffusion axis. The proposed CVs have three remarkable properties: (i) they are simple (Figure 3A), (ii) they allow constrained ligand motion to the volume of the cavity (Figure 3B), and (iii) their low dimensionality allows exploration of the CVs space in reduced simulation times. CV1 and CV2 share the spirit of the well-known path CVs [49], a system that uses orthogonal collective variables to define the progression of the system along a putative path. However, this approach requires a guess of the possible path, while the CVs proposed in this work only require a guess of a privileged axis of space.

Upon establishing proper collective variables, the well-tempered metadynamics (WT-Meta) algorithm [32] was selected as the method to explore the energy landscape. Bias temperature, a specific WT-Meta parameter that conditions the explored energy landscape, was fixed at 4500K after testing different values. The final setup included both a lateral barrier and a transversal barrier at the limits of the diffusion axis, as described in Figure 3A, to limit diffusion of the ligand into the solvent. The position of these barriers was established upon visual inspection of the solvated ligand in simulations in which the ligand had escaped into the solvent.

Energy landscapes revealed by this method are smooth and have well-defined minima around the docking starting position, a result which must be interpreted as a sign of the efficacy of docking methods. The natural ligand in the orientation A presents two basins identified as A_0_ and A_1_, separated by a small transition state, indicating an equilibrium between states (Figure 5). The landscape of the natural ligand in orientation B also shows two basins (B_0_ and B_1_), with the docking position being the most stable (B_0_). The second basin has its minimum inside the cavity, at a location where the ligand would be exposed to solvent (Figure 5). The number of contacts between the protein and the ligand remains as high as in the bound state, so there is an inner position in which the ligand is also protected and stable, although to a lesser extent.

As for possible paths, our results indicate that the ligand will not take the path from A_0_ to L (Figure 5) since that would mean exiting through a large conformational barrier whose energy exceeds the limits of the exploration at the bias temperature employed. On the other hand, the ligand in orientation B can exit by two different paths. The first path would require a two-step process in which first the transition from B_0_ to B_1_ takes place, and then a second transition from B_1_ to B_R_, which seems to have lower energy than its homolog, B_L_. The second path would only have one step, since B_L_ does not seem to form any basin and behaves like a saddle point. Thus, the path B_L_ to B_0_ is more likely.

Gathering our free energies results and tunnel diffusion, one may conclude that the most likely pose and path is orientation B and the path from B_0_ to B_L_, respectively. This finding is in complete agreement with the experimental work of Shenkarev et al. [10], who demonstrated that several lipids harbored in the cavity of lentil LTP present the geometry which we call orientation B. However, our results would also be compatible with possible diffusion along an opposite path, although with slower dynamics.

This article proposes a new computational protocol that can be applied to systems with the following properties: (i) being protein–ligand complexes, (ii) having a tunnel-shaped cavity, and (iii) allowing ligand motion along a plausible diffusion axis. Although these properties are obviously not general in protein–ligand complexes, they are present in a large set of proteins that carry ligands, especially of lipidic nature, such as LTPs. This protocol could be summarized in the following steps: (1) pocket detection (using dynamic or static information), (2) variance decomposition to obtain a diffusion axis, (3) well-tempered metadynamics simulations, and (4) analysis of the results. Examples of input for well-tempered metadynamics and source code for the analysis of the output can be found at (GitHub repository: URL upon publication).

To fully understand the processes accompanying the motion of a ligand inside a protein cavity, further studies and new simulations are obviously necessary. First, a funnel metadynamics could be helpful to determine free energy and to test the robustness of predictions in this work. Second, since lipid uptake in a protein, such as the LTP studied here, could take place in the membrane–solvent interphase, similar MD simulations in conditions of membrane adsorption would permit obtaining the energy landscape of their own lipid uptake. In order to extend the scope of this research to integrate it into the molecular mechanisms of lipid transport and protein recognition associated with the responses to the presence of allergen proteins carrying their lipid cargo, it would also be interesting to conduct that study in other allergenic and non-allergenic LTPs. This could help assess the different behaviors of otherwise similar proteins in the sensitization phase of food allergy.

## 4. Materials and Methods

### 4.1. Protein Structure

The structure of Pru p 3 was obtained from the Protein Data Bank, entry “2ALG”, that corresponds to an X-ray structure at a resolution of the electron density map of 2.3 Å [9]. The original model contains a crystallographic dimer where each unit is bounded to a different ligand and has some minor structural changes regarding rotamers of some residues. Chain B, ligands, and crystallographic waters were removed to set the initial structure of Pru p 3.

### 4.2. Binding Poses of Ligands

Ligands were first modeled using Chimera Build Tools and then optimized at repeated cycles of steepest-descent and conjugate-gradients minimizations with Chimera [50]. First, the natural ligand of Pru p 3 was constructed, and the geometry of its complex with the LTP structure was then obtained in orientations A and B using Autodock Vina in Chimera with default parameters in a user-defined box that covered all the volume of the protein tunnel. The poses with the lowest protein–ligand affinity energy in Autodock Vina calculations were selected in all cases without modifying its default parameters. The initial binding poses of phytosphingosine and sphingosine in the two orientations of their complexes with Pru p 3 were obtained in the same manner, after remodeling the natural ligand with Chimera to keep only the sphingoid tail.

### 4.3. Force Field Parametrization

CHARMM 3.6 force field parameters were used for protein, water, and ions. Parametrization of the three ligands relied on the CGenFF parametrization by analogy with setup of parameters and input files for force field calculations prepared using the web server CHARMM-GUI [51]. Input for parametrization of the protein only required to define the four disulfide bonds Cys13-Cys27, Cys28-Cys73, Cys3-Cys50, and Cys48-Cys87 that are highly conserved in the LTP family. Parametrization of ligands required uploading separate files with their optimized geometries in Mol2 format, obtained via OpenBabel [52] from their corresponding PDB files.

### 4.4. Molecular Dynamics

Molecular dynamics calculations were performed using NAMD 2.13 [53] and CHARMM 3.6 force field [54]. Proteins were immersed in 3D solvation boxes of different sizes using the TIP3P model of water and sizes, adjusted in each case to allow stable simulations in the NPT ensemble. Sodium and chloride ions were added to counter the protein charge while setting a final salt concentration of 0.150 M. The particle–mesh Ewald summation method was used for long-range electrostatic interactions and a 10Å cut-off was set for the short-range non-bonded interactions. The temperature was set to 298K and the pressure was set to 1atm.

The conventional three-step molecular dynamics treatment was applied in all cases: 1—initial minimization of the complete system to guarantee that the starting point of the simulation is a minimum of the potential energy; 2—equilibration of water to provide the solvent the temperature of the simulation; and 3—production runs of MD simulations with 50 million steps at 2fs time step, i.e., trajectories over a simulation time of 100 ns. Langevin dynamics for temperature control and Noose–Hoover Langevin piston method for pressure control were used.

### 4.5. Map of Interacting Residues

The natural ligand of Pru p 3 was split in three parts: head (camptothecin moiety), tail (phytosphingosine chain moiety), and bridge (polar segment linking both moieties). All distances from all atoms in each protein residue to the atoms of each part of the ligand were calculated and used as input to a logistic function spanning the range –1 to 1. The values calculated along time and for all residues were used to construct a covariance matrix. Finally, a hierarchical clustering was performed over the covariance matrix after inspection of the dendrograms. For ease of comparison, four clusters were used for the natural ligand of Pru p 3 in the two orientations A and B. Details on this analysis can be found at one of the notebooks at the GitHub repository, URL https://github.com/brunocuevas/exploring_prup3_landscapes.

### 4.6. Free Energy Estimation

Initial free-energy estimations were determined using a hybrid methodology to obtain both the enthalpy and entropy terms. Since MD simulations showed that the ligand was stabilized inside the cavity, these calculations were performed using the 500 last frames of the trajectory file for each simulation (representing around 20 ns). The ΔH was computed using the Molecular Mechanics Poisson–Boltzmann Surface Area (MM–PBSA) approximation implemented in the CaFE plugin for VMD [55]. ΔS was obtained using a PCA-histogram-based approximation to compute the distribution of probabilities of the microstates, implemented in the CARMA package [56]. Then, Gibbs free energies were calculated as ΔG = ΔH − TΔS at T = 298 K. These calculations were repeated 10 times over the same dynamics in order to provide an estimate of the intrinsic variance of the method.

### 4.7. Collective Variables Setting

MDpocket [35] was used to study the fluctuations of the protein cavity along MD trajectories. Ligands were not included in the calculation of pockets. From the volumetric file of frequencies, all grid points with a pocket-frequency over an arbitrary value (0.2) were chosen. Then, major covariance axes were obtained by eigenvalue decomposition of the covariance matrix of the coordinates. Since axes were similar for the possible poses of ligands, the same axes were considered for the three ligands studied. This decomposition gave place to a system with two collective variables: (i) a distance along the major axis of the tunnel-like cavity of Pru p 3, and (ii) a distance orthogonal to the major axis.

To maintain the axes relative to the protein coordinates, thus avoiding the bias of molecular diffusion and tumbling, coordinates were made relative to a set of points of the protein structure, so collective variables were always calculated in the roto-translational frame that minimizes the RMSD between the coordinates of each time step and those of the initial reference structure. This set of points were selected as the alpha carbons of residues whose RMSF obtained from MD trajectories were below a certain threshold, a criterion which mostly selected the α-helices of Pru p 3. Collective variables were calculated over the center of mass of carbon atoms of aliphatic chains of the ligand. The camptothecin head segment of the natural ligand of Pru p 3 was not included in the CVs calculation, as it would introduce artifacts due to the torsions of that head, which was considered uninteresting in the ligand diffusion process. The origin for all collective variables was set at the CVs values at the starting point.

### 4.8. Metadynamics

Metadynamics simulations were performed using the plugin Colvars [57], integrated into NAMD. This plugin offers a wide variety of methods to bias simulations. Metadynamics in the well-tempered implementation was used to explore a single degree of freedom (the diffusion axis, CV1). Harmonic walls were used to avoid diffusion of ligands into the solvent, which would lead to slow convergence. These harmonic walls were set around the cylinder that covers the cavity volume, and at the covers (Figure 3). Parameters are specified in Table 2. Mathematical expressions concerning the harmonic wall bias and the well-tempered metadynamics bias are presented in Appendix A.

### 4.9. Analysis

Results were analyzed using the Colvars module of VMD [58] and the Python package Prody [59]. The analysis was performed in an IPython notebook available at (GitHub repository: URL upon publication). The Essential Dynamics module of the Prody package was especially useful for processing our results.

## Figures and Tables

**Table 1:** Estimates of protein–ligand binding free energies, enthalpies, and entropies at 298 K.

Ligand	Orientation	ΔG_bind_ (kcal/mol)	ΔH (kcal/mol)	TΔS (kcal/mol)
Natural Ligand	A	−7.27 ± 0.59	−18.63 ± 0.68	−11.36 ± 0.23
Natural Ligand	B	−11.15 ± 0.58	−23.03 ± 0.49	−11.88 ± 0.26
Phytosphingosine	A	−10.20 ± 0.41	−21.73 ± 0.38	11.53 ± 0.19
Phytosphingosine	B	−3.33 ± 0.49	−16.07 ± 0.47	−12.75 ± 0.18
Sphingosine	A	−6.09 ± 0.59	−19.02 ± 0.59	−12.93 ± 0.20
Sphingosine	B	−9.36 ± 0.33	−22.48 ± 0.27	−13.12 ± 0.23

**Table 2:** Parameters of the metadynamics simulation for each of the bias.

Type of Bias	Variable	Parameters
Harmonic wall	CV2	K = 40.0 kcal/Å·mol, s_0_^−^ = −20 Å, s_0_^+^ = 12.5 Å
Harmonic wall	CV1	K = 40.0 kcal/Å·mol, s_0_^−^ = 0 Å, s_0_^+^ = 9.0 Å
WT-Metadynamics	CV1	H = 0.1 kcal/Å·mol, δ = 0.05 Å, Δ T = 4500 K, τ_G_ = 1000 fs

## Supplementary Materials

The following supplementary materials can be found at https://www.mdpi.com/1422-0067/20/6/1432/s1.

## Abbreviations

CV	Collective variable
LTP	Lipid transfer protein
MD	Molecular dynamics
PCA	Principal components analysis
RMSD	Root mean squares deviation
RMSF	Root mean squares fluctuation

## Appendix A

Two biases were used in enhanced sampling methods: the harmonic walls bias and the well-tempered metadynamics bias. Harmonic walls are defined as a harmonic potential that only applies when the biased CV, hereby called “s”, is out of a given range [s_0_^−^ s_0_^+^].

(A1) $$V_{hw}\left(s\right)=\frac{1}{2}k{\left(s-s_{0}^{-}\right)}^{2}\;only\;if\;s<s_{0}^{-}$$

(A2) $$V_{hw}\left(s\right)=\frac{1}{2}k{\left(s-s_{0}^{+}\right)}^{2}\;only\;if\;s>s_{0}^{+}$$

The metadynamics bias is a sum of gaussians centered along sampled points of the trajectory of the biased collective variable.

(A3) $$V_{meta}\left(s\left(t\right),t\right)=\underset{t^{\prime }=n\tau_{G}\;t^{\prime }<t}{\sum }he^{-\frac{{\left(s\left(t\right)-s\left(t^{\prime }\right)\right)}^{2}}{2\delta^{2}}}$$

In this expression, τ_G_ is the deposition time interval, δ is the width of the added gaussians, and *h* is their height. In the case of the well-tempered metadynamics, the constant height *h* is substituted by an underlying potential-dependent variable, *h’*.

(A4) $$h^{\prime }=he^{-\frac{V\left(s,t\right)}{\Delta T}}\tau_{G}$$

In this equation, gaussian height is confined in the range (*h*, 0).
