# Supplementary material for: Energy Landscapes of Ligand Motion Inside the Tunnel-Like Cavity of Lipid Transfer Proteins: The Case of the Pru p 3 Allergen
Source: Int J Mol Sci. 2019 Mar 21;20(6):1432. doi: 10.3390/ijms20061432 (PMC6471300; doi:10.3390/ijms20061432)
Supplement: Supplementary file 1 [file ijms-20-01432-s001.pdf]

Energy landscapes of ligand motion inside the tunnel-like cavity of lipid transfer proteins: the case of the Pru p 3 allergen

Bruno Cuevas-Zuviría, María Garrido-Arandia, Araceli Díaz-Perales and Luis F. Pacios

Supplementary Material

Contents

		Page
**Figure S1.**	RMSD of Pru p 3 protein and complete ligands	S2
**Figure S2**.	RMSD of Pru p 3 and its natural ligand in 3 replicates of trajectories	S3
**Figure S3A**.	Map of clustered residues for the natural ligand in orientation A	S4
**Figure S3B**.	Map of clustered residues for the natural ligand in orientation B	S4
**Table S1.**	Estimates of protein-ligand binding free energies obtained in three repeats of 100-ns MD simulations of the Pru p 3-ligand complex	S5
**Figure S4.**	Diagram of estimates in Table S1	S5
**Figure S5.**	Initial and final structures after 100-ns MD simulations of complexes of Pru p 3 with the three ligands in orientations A and B	S6
**Figure S6**.	Histogram of the radius of gyration vs. CV1 for orientation A	S7
**Figure S7**.	Histogram of the radius of gyration vs. CV1 for orientation B	S8
**Figure S8**.	Number of contacts between Pru p 3 and its natural ligand vs. CV1 for orientations A and B	S9

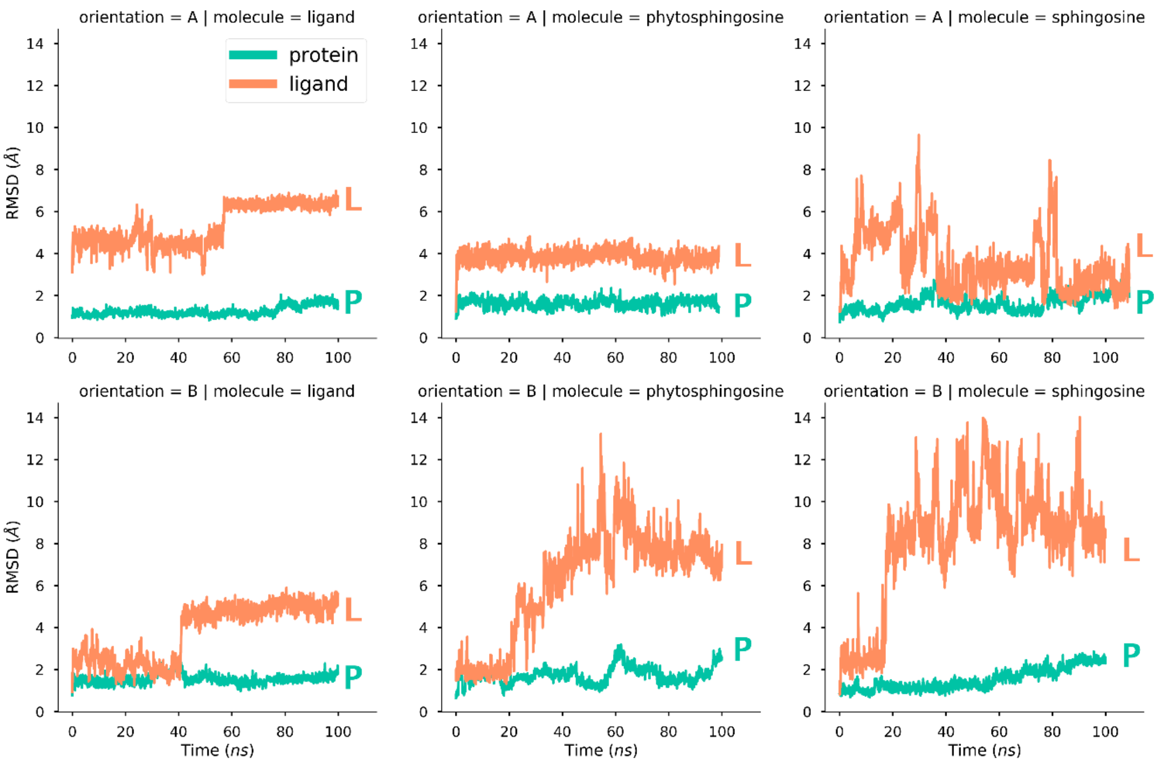

Figure S1. RMSD of protein (backbone atoms) and ligands along 100-ns trajectories for the orientations A and B inside the cavity of Pru p 3 of the three ligands studied: the complete structure of the natural ligand of Pru p 3 ("ligand"), phytosphingosine, and sphingosine. The image below illustrates the geometry for the transition observed in the RMSD curve for the ligand of Pru p 3 in orientation B at ~40 ns. This transition corresponds to a torsion around the segment that links the camptothecin polar moiety (largely exposed to the solvent) and the phytosphingosine tail that remains harbored in the tunnel-like cavity.

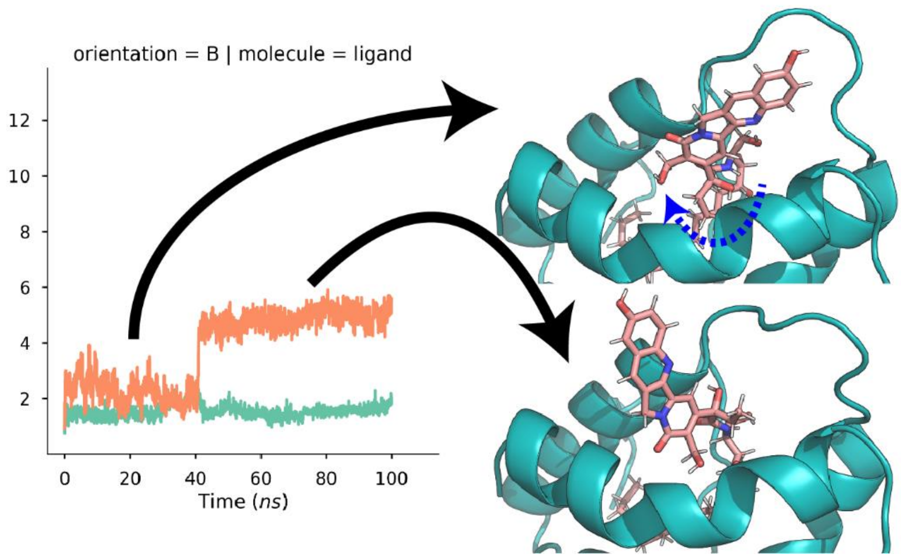

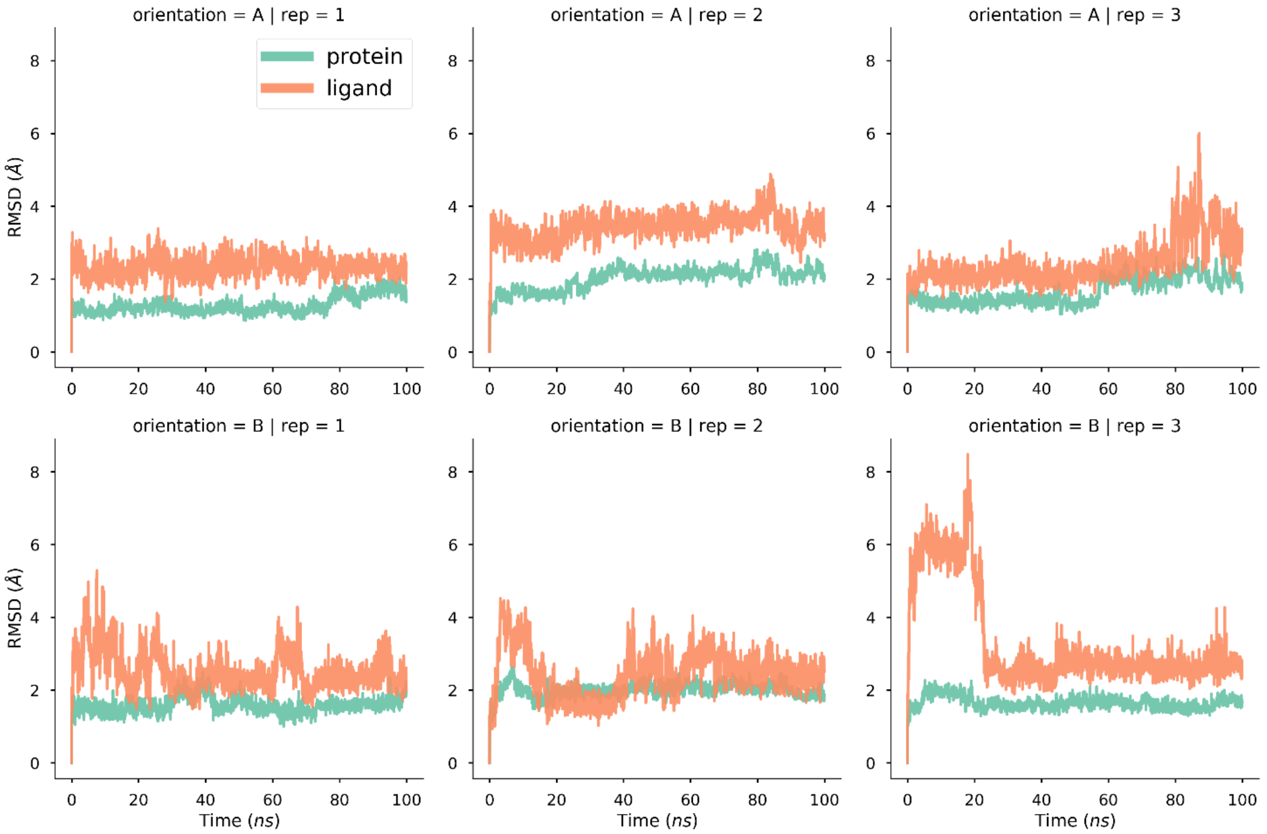

Figure S2. RMSD of protein (backbone atoms) and phytosphingosine tail of the natural ligand of Pru p 3 for orientations A and B inside its cavity along three replicates of 100-ns MD trajectories.

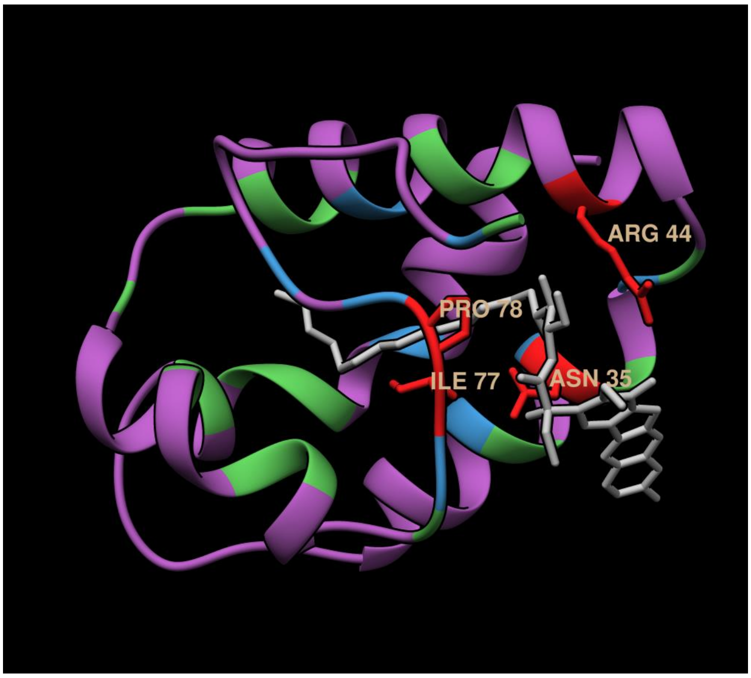

Figure S3A. Residues belonging to each of the clusters found with the contact-covariance approach described in notebooks available at the GitHub repository, URL https://github.com/brunocuevas/exploring_prup3_landscapes for the natural ligand of Pru p3 in the orientation A. Residues shown in sticks form the cluster with more constant interaction with the ligand along the MD trajectory. Locations in red and blue indicate two clusters of residues that interact with the segment of the ligand that joins the polar head and the aliphatic chain. Locations in green represent interactions with the non-polar aliphatic chain.

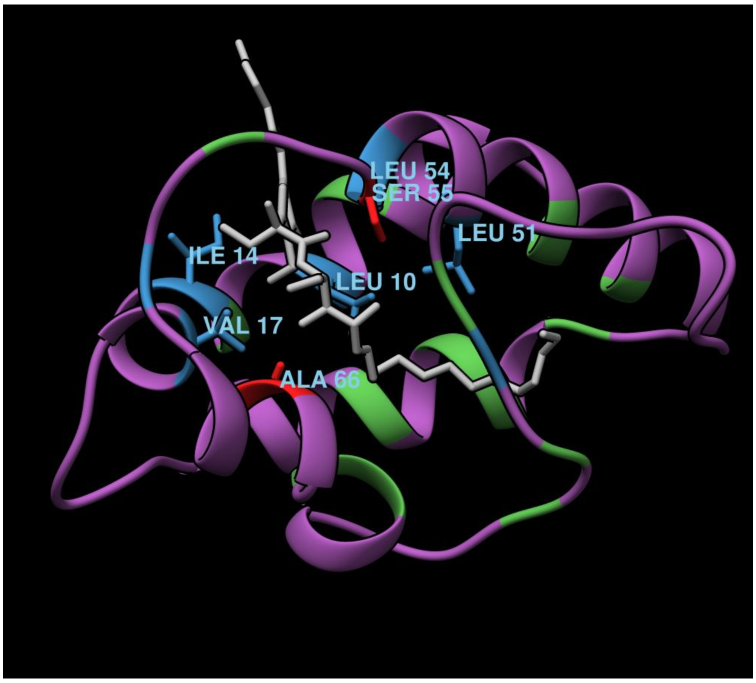

Figure S3B. Same as Figure S3A for the natural ligand of Pru p3 in the orientation B.

Table S1. Estimates of protein-ligand binding free energies, enthalpies and entropies at 298 K obtained after three repeats of 100-ns MD simulations of the complex between Pru p 3 protein and its natural ligand in both orientations A and B. The corresponding values for each simulation are also given.

Orientation	ΔG_{bind}	ΔH	$T\Delta S$	Rep	ΔG_{bind}	ΔH	$T\Delta S$
A	-5.55 ± 1.45	-18.60 ± 0.76	-13.04 ± 1.24	1	-7.27 ± 0.59	-18.63 ± 0.68	-11.36 ± 0.23
				2	-5.12 ± 0.70	-18.89 ± 0.64	-13.76 ± 0.35
				3	-4.28 ± 0.82	-18.29 ± 0.90	-14.01 ± 0.23
B	-11.21 ± 1.42	-23.29 ± 1.51	-12.07 ± 0.29	1	-11.15± 0.58	-23.03 ± 0.49	-11.88 ± 0.26
				2	-12.81±0.51	-25.12 ± 0.44	-12.31 ± 0.21
				3	-9.69±0.82	-21.73 ± 0.69	-12.04 ± 0.22

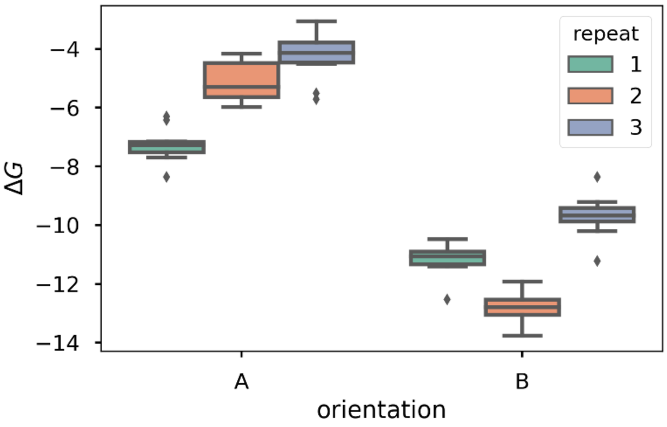

Figure S4. Diagram of estimates of Pru p 3/natural ligand binding free energies obtained in three repeats of 100-ns MD simulations (values in Table S1).

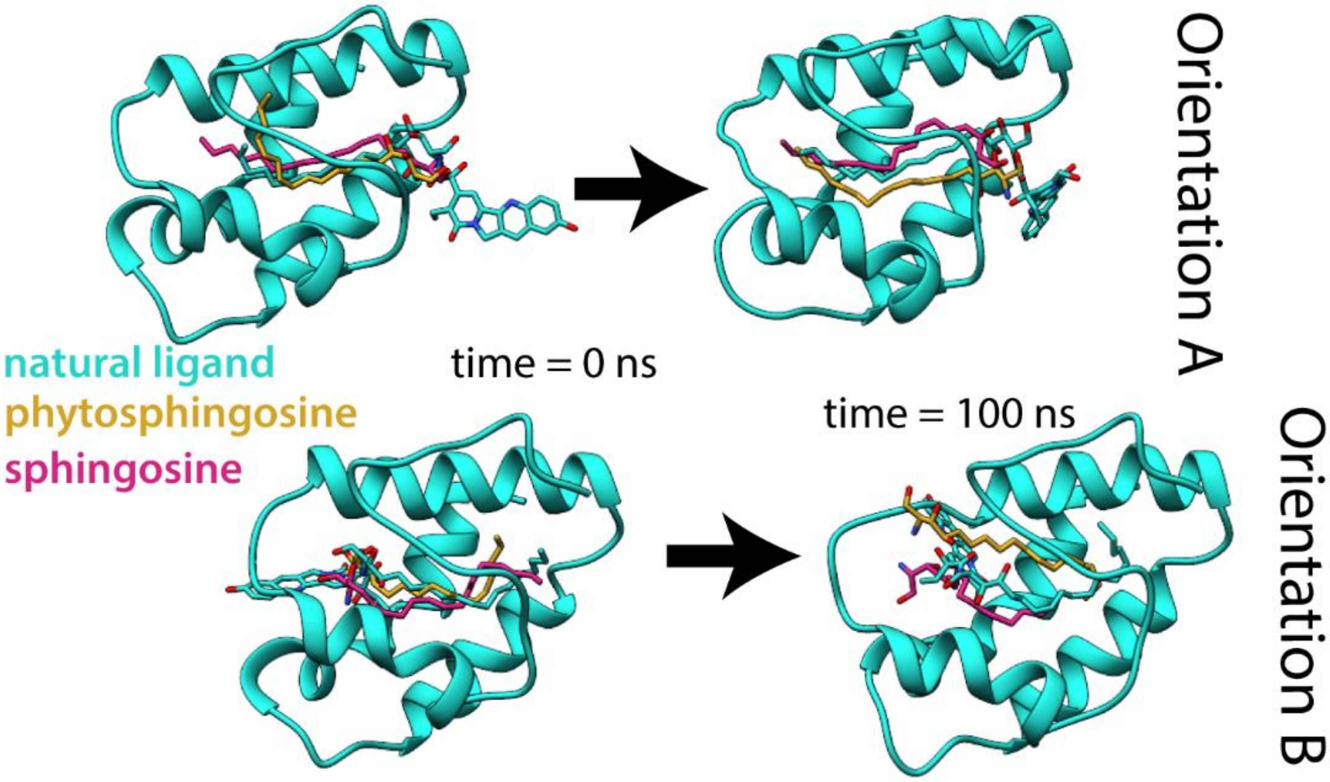

Figure S5. Initial (0 ns) and final structures after 100-ns MD simulations of complexes of Pru p 3 with the three ligands studied: its natural ligand, phytosphingosine, and sphingosine in orientations A and B. For ease of comparison to highlight the geometries of ligands, only a ribbon image of Pru p 3 protein resulting from the superposition of backbones in the three complexes is shown in each frame.

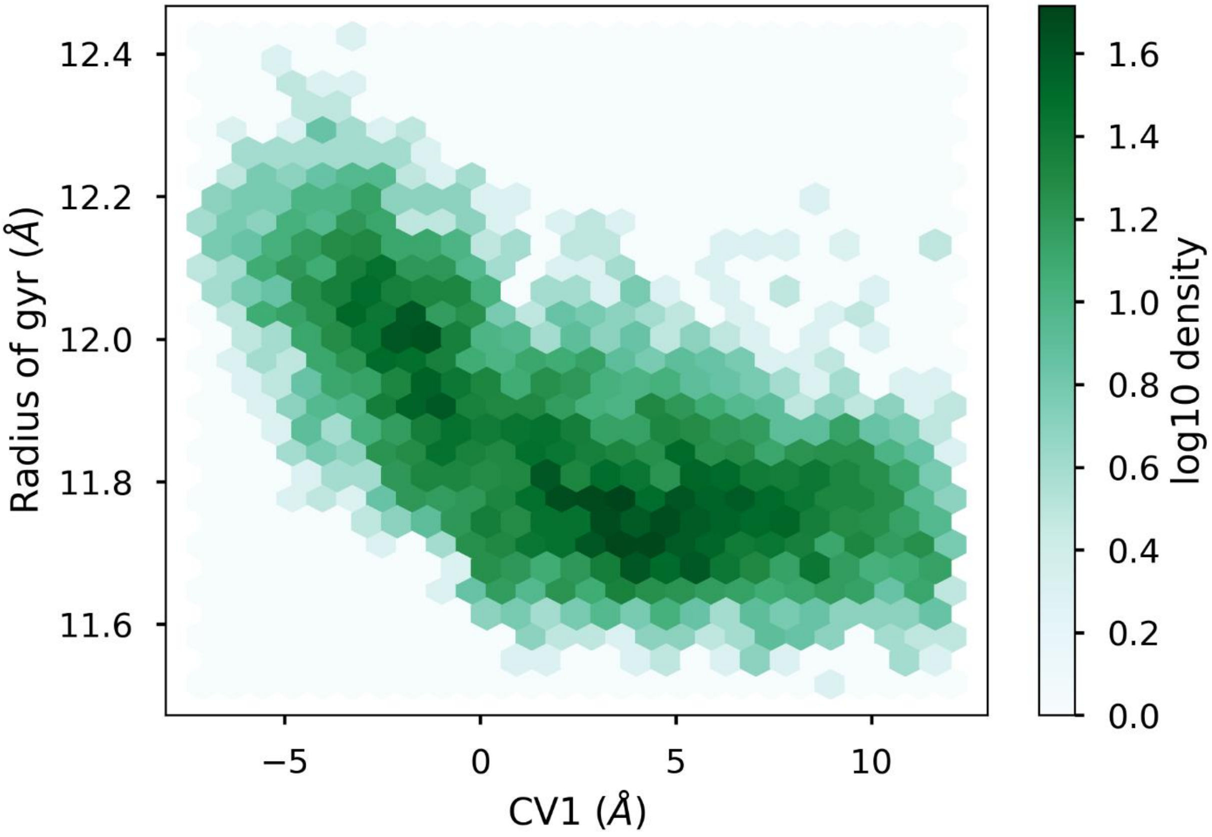

Figure S6. Histogram of the radius of gyration of Pru p 3 protein computed with Cα atoms vs. collective variable CV1 for the natural ligand of Pru p 3 inside its tunnel-like cavity in the orientation A. The trend change from -5 Å to 0 Å is related with the polar head of the ligand getting in part inside the cavity.

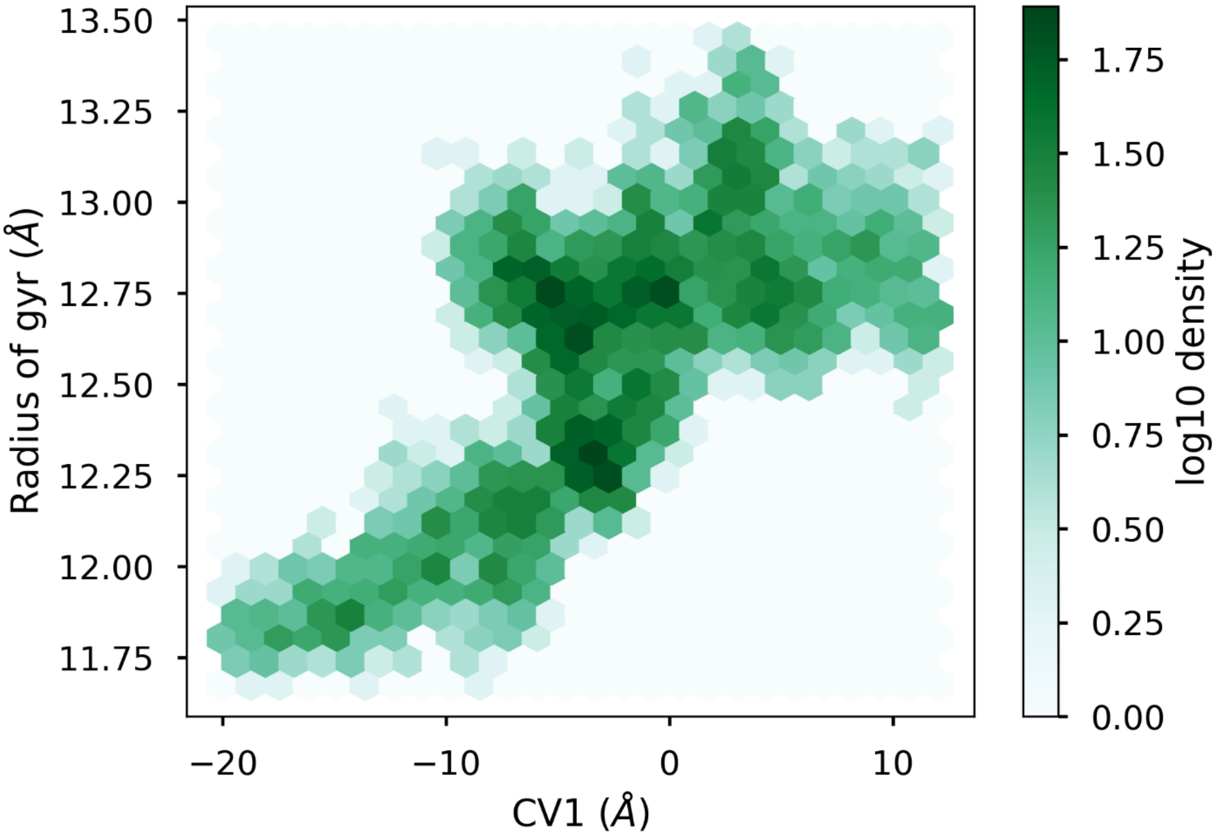

Figure S7. Histogram of the radius of gyration of Pru p 3 protein computed with Cα atoms vs. collective variable CV1 for the natural ligand of Pru p 3 inside its tunnel-like cavity in the orientation B. A similar trend to that shown in Fig. S6, attributable to the partial entrance of the polar head of the ligand inside the cavity is also observed. However, the relation is now less homogeneous and the radius of gyration is greater than in Fig. S6.

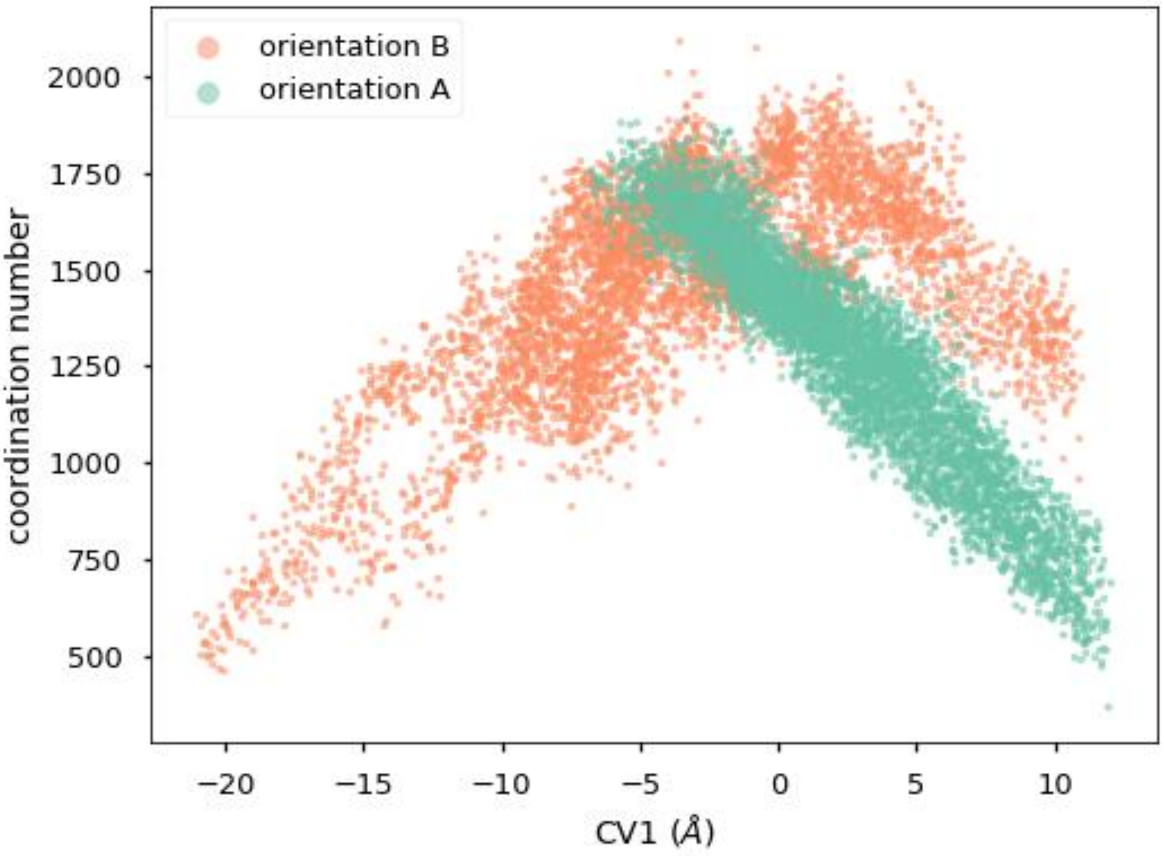

Figure S8. Number of contacts between Pru p 3 protein and its natural ligand vs. collective variable CV1 calculated with the Colvars module of VMD (see Colvars manual at https://github.com/Colvars/colvars) for both orientations A and B of the natural ligand inside the tunnel-like cavity of Pru p 3. Orientation A shows a linear trend only in the region from -5 Å to 0 Å whereas orientation B shows a peak near 0 Å and then a clear decrease thereafter.
